# Breakthrough Infections: A Challenge towards Measles Elimination?

**DOI:** 10.3390/microorganisms10081567

**Published:** 2022-08-04

**Authors:** Clara Fappani, Maria Gori, Marta Canuti, Mara Terraneo, Daniela Colzani, Elisabetta Tanzi, Antonella Amendola, Silvia Bianchi

**Affiliations:** 1 Department of Health Sciences, Università degli Studi di Milano, 20146 Milan, Italy; 2 Department of Clinical Sciences and Community Health, Università degli Studi di Milano, 20122 Milan, Italy; 3 Department of Pathophysiology and Transplantation, Università degli Studi di Milano, 20122 Milan, Italy; 4 Coordinated Research Center “EpiSoMI”, Università degli Studi di Milano, 20133 Milan, Italy

**Keywords:** measles, breakthrough infection, measles vaccine, vaccination failures

## Abstract

Measles is one of the most contagious diseases known to man. Despite the existence of a safe and effective live attenuated vaccine, measles can appear in vaccinated individuals. Paradoxically, breakthrough cases increase as vaccination coverage in the general population rises. In measles endemic areas, breakthrough cases represent less than 10% of total infections, while in areas with high vaccination coverage these are over 10% of the total. Two different vaccination failures have been described: primary vaccination failure, which consists in the complete absence of humoral response and occurs in around 5% of vaccinated individuals; and secondary vaccination failure is due to waning immunity or incomplete immunity and occurs in 2–10% of vaccinees. Vaccination failures are generally associated with lower viral loads and milder disease (modified measles) since vaccination limits the risk of complicated disease. Vaccination failure seems to occur between six and twenty-six years after the last vaccine dose administration. This review summarizes the literature about clinical, serological, epidemiological, and molecular characteristics of measles breakthrough cases and their contribution to virus transmission. In view of the measles elimination goal, the assessment of the potential decline in antibody protection and the targeted implementation of catch-up vaccination are essential.

## 1. Introduction

Measles is one of the most contagious diseases known to humankind, with a basic reproduction number (R_0_) assumed to be between 12 and 18. It spreads through coughing and sneezing, close personal contact, or direct contact with infected nasal or throat secretions. The disease is typically characterized by an erythematous and blotchy red (maculopapular) rash that starts from the face, upper neck, and behind the ears and then spreads to the trunk reaching hands and feet. The rash lasts for 3–4 days and then fades, disappearing from the face first [1,2]. Most measles-related deaths are caused by complications associated with the disease, such as: blindness, encephalitis, severe diarrhoea and related dehydration, ear infections, or severe respiratory super-infections such as pneumonia [1].

Measles is caused by measles virus (MV). MV is a single-stranded, negative-sense, enveloped, non-segmented RNA virus belonging to the species *Measles morbillivirus* of the genus *Morbillivirus*, within the family *Paramyxoviridae*. MV genome consists of 15,894 nucleotides and encodes eight proteins. The non-structural proteins V and C are involved in the cellular response to infection. The hemagglutinin (H), essential for viral binding to cellular receptors, and fusion (F) proteins mediate fusion of the viral envelope with the host cell membrane and are the main targets of neutralizing antibodies. Together with the matrix protein (M), which is important for virus assembly, they represent the three membrane-associated proteins. The ribonucleoprotein (RNP) complex consists of the nucleocapsid (RNA bound to RNA-binding nucleocapsid proteins N) with a polymerase-associated phosphoprotein (P), and a large protein (L, including an RNA-directed RNA polymerase (RdRP), mRNA guanylyl- and methyltransferases, and methylation functions required for the capping of mRNAs) [2,3]. MV is related to several viruses infecting animals (e.g., Canine Distemper Virus, Rinderpest virus) and humans are its natural host [2].

According to the World Health Organization (WHO) guidelines, measles cases classification should be conducted according to clinical, epidemiological, and/or laboratory criteria. Any individual presenting with fever, maculopapular rash lasting 3 or more days, and at least one additional symptom among cough, coryza, or conjunctivitis fulfills clinical criteria for measles. Epidemiological criteria are met when contact tracing evidences an epidemiological connection that could have been the source of interhuman transmission. Laboratory criteria must meet at least one of the following parameters: isolation of MV from a clinical specimen (throat or nasopharyngeal swabs, nasal aspirates, or 10 to 50 mL of urine, collected as soon as possible after the rash onset [4]), detection of MV nucleic acid in a clinical specimen (the same samples collected for isolation can be used, as well as oral fluids and dried blood [4]), identification of anti-MV specific antibody response characteristic of the acute infection (1–10 days) in serum or saliva, or detection of MV antigen by a direct fluorescent assay in a clinical specimen (nasopharyngeal and throat swabs, and urine specimens [5]) using MV specific monoclonal antibodies. Laboratory results need to be interpreted according to the vaccination status, and specific virological investigations (i.e., the detection of a wild viral strain) are required to confirm the diagnosis in recently vaccinated patients (see the following sections for more details) [6].

The exclusively interhuman transmission, the absence of healthy carriers, and the availability of a safe, effective, and easily administered vaccine make measles an eradicable disease [7]. Since 2010, the WHO, established three milestones critical towards the future eradication of measles: increase routine coverage with the first dose of measles-containing vaccine by more than 90% nationally and more than 80% in every district; reduce and maintain annual measles incidence to less than five cases per million; and reduce estimated measles mortality by more than 95% from the 2000 estimate [1]. In 2012, the Health Assembly endorsed the Global Vaccine Action Plan, which included the objective of eliminating measles in four WHO regions by 2015 and in five regions by 2020 [8]. Nonetheless, despite a total of 82 countries were verified as having eliminated measles at the end of 2018, and the enormous progress made in implementing surveillance and increasing vaccination coverage with a sizeable reduction in the measles diseases burden, the regional elimination target provided by the Global Measles and Rubella Strategic Plan 2012–2020 [9] has not yet been achieved. Globally, the number of reported measles cases has more than doubled from 2017 to 2018, from 170,000 to 350,000, and the increasing trend continued into 2019, with several countries experiencing large measles outbreaks. A new goal of measles elimination has been defined with the 2021–2030 strategic framework [10].

## 2. Measles Vaccines and Immunization Programs

Several attenuated measles vaccines are available worldwide, either as single-virus vaccines or in combination with the rubella and mumps vaccines (MMR) or with the rubella, mumps, and chickenpox vaccines (MMRV). Although there are 24 recognized MV genotypes, MV is considered serologically monotypic [11] and most of the available vaccine strains derive from the Edmonston strain, isolated in 1954 [12]. The principal target of human antibodies is the H protein. Sequence analyses of the H gene performed in several studies did not show specific mutations associated with immune escaping, and this antigenic stability could be at the basis of the effectiveness of the present vaccine [13,14,15,16]. Likewise, no significant differences have been found in strains circulating in vaccinated or not vaccinated individuals [17,18].

The measles vaccine induces both humoral and cellular immune responses and antibodies appear between 12 and 15 days after vaccination, peaking at 21 to 28 days [2]. Measles immunization programs consist of the administration of two doses of measles vaccines. Although there are variations between vaccination calendars of the various countries, according to WHO recommendations for routine immunization, the first dose is usually given at the age of approximately nine months in countries with ongoing measles transmission, in which the risk of measles mortality remains high, and, to take advantage of the higher seroconversion rates achieved at an older age, at the age of 12 months in countries with low levels of measles transmission. The second dose should be administered at 15 to 18 months of age or at school entry [19,20]. However, every opportunity should be taken to vaccinate all children that missed one or both routine doses [20].

Both MV infection and vaccination are likely to confer lifelong immunity against the disease. During the 1846 Faroe Island epidemic, the Danish physician Peter Panum noted that people who had measles in the 1781 epidemic did not develop signs of the disease [21]. Furthermore, the study of the live Edmonston B measles vaccine in the 1950s–1960s showed post immunization antibody decay curves similar to those of people who had had the natural infection [22]. According to the Centers for Disease Control and Prevention (CDC), one dose of MMR vaccine is 93% effective against measles while two doses are 97% effective [23]. Two doses of the vaccine are recommended to ensure immunity and prevent outbreaks since about 15% of vaccinated children fail to develop immunity from the first dose [1].

It was estimated that before the introduction of the vaccine in 1963, measles caused 2.6 million deaths each year. Routine vaccination for children and mass immunization are key public health strategies to reduce global measles deaths [1]. Nonetheless, vaccination coverage remains low or very low in several countries. In 2019, the coverage for the first dose of measles-containing vaccines was below 50% in seven countries, and for the other 23 it was below 70%, indicating that 30–50% of children in these countries had not received any doses of measles vaccine through routine service delivery mechanisms [10]. To achieve measles elimination and eradication, vaccination coverage for two vaccine doses >95% is necessary. However, until 2020, the worldwide vaccination coverage for one and two doses were 84% and 70%, respectively. The WHO region with the highest percentage of fully vaccinated individuals was the Western Pacific Region (94%), followed by European Region (91%), Eastern Mediterranean Region (76%), Region of the Americas (73%), and African Region (36%) [24]. Although in the 2020–2021 period, the containment measures implemented against the COVID-19 pandemic significantly reduced MV circulation, giving the opportunity to accelerate progress towards measles elimination, all six WHO Regions reported a disruption in immunization activities that led to an accumulation of susceptible individuals [25]. Indeed, in the first two months of 2022 worldwide reported measles cases increased by 79%, compared to the same period in 2021 [26].

## 3. Measles Virus Infection and Vaccine Failure

Despite the availability of a safe and highly effective live attenuated vaccine, measles can manifest in individuals with a documented vaccination history. During the Cincinnati and St. Louis epidemics of 1971–1973, Cherry et al., and Plotkin et al., described for the first time the existence of measles vaccination failure cases [27,28,29]. Indeed, nowadays it has been observed that the duration of protection conferred by measles vaccine is more variable and shorter than that acquired through measles infection, with an estimated 5% of children losing protective antibody titres 10–15 years after vaccination [2,18,30]. Breakthrough cases, which occur when a person becomes sick with a disease despite having received the vaccine for that disease (vaccine failure), do play an important role in the epidemiology of the disease.

**Figure 1:**
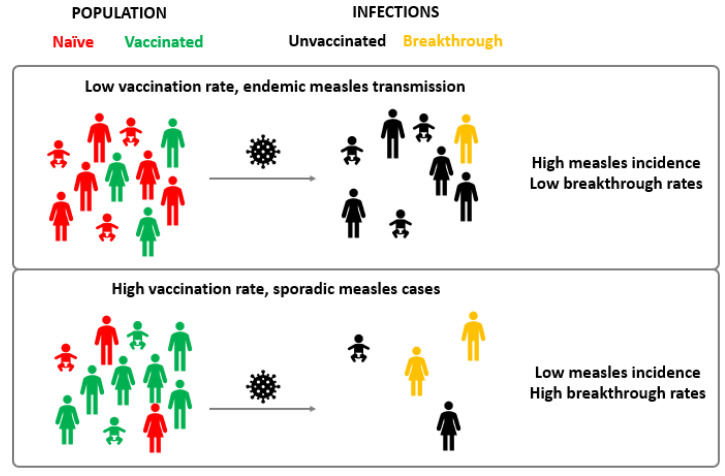
Measles breakthrough cases in countries with endemic or sporadic measles transmission. In populations where the majority of individuals are naïve, MV can circulate endemically and most of the infected individuals will be unvaccinated subjects (**top**). On the other hand, in populations with high vaccination coverages (and lower MV circulation), the number of vaccination failure cases among susceptible individuals will be higher and so will be the proportion of breakthrough cases (**bottom**).

As vaccination coverage increases in the general population, a proportional increase in the frequency of measles cases among vaccinated individuals is expected, as long as MV circulates [31]. This happens because, with fewer non-vaccinated individuals, most of the susceptible subjects are those that did not develop a protective immune response after vaccination (Figure 1). Indeed, the portion of breakthrough cases over the total of measles infections is higher in countries with high vaccination coverage. For example, between 2000 and 2015, California registered 400 cases of laboratory-confirmed measles and 20% were in vaccinated individuals [32]. In the post elimination phase (2014–2020), Spain observed a portion of breakthrough cases of 14%, which was a substantial increase compared to the 2003–2014 period, during which around 3.5% of confirmed cases had received two doses of MMR vaccine [33,34]. Sundell et al., described an outbreak that occurred in Gothenburg, Sweden, in 2017 during which 16 of the 28 confirmed cases of measles were breakthrough infections [35]. The percentages of breakthrough cases identified in various studies in different countries according to the level of measles circulation are reported in Table 1.

Additionally, regardless of whether they are produced as a result of the disease or the vaccine, antibodies against MV seem to decay more rapidly in populations where disease prevalence is lower [30,32,36] and/or in isolated populations. This occurs because people do not get exposed to the wild virus and fail to receive a natural booster [37]. In other words, in areas with a higher vaccination coverage MV circulation is reduced, and this corresponds to a faster antibody decay due to the absence of repeated immune system stimulations. This results in an increased chance for vaccinated individuals to acquire the infection.

**Table 1:** Percentage of breakthrough measles cases according to the level of MV circulation.

Study	Measles Circulation Level	Study Period	Region/Country	Breakthrough Cases (%)
Cherry et al. (2018) [32]	Post elimination	2000–2015	California	20
Sundell et al. (2019) [35]	Post elimination	2017–2018	Gothenburg, Sweden	57
Augusto et al. (2019) [38]	Post elimination	2017	Portugal	37
López–Perea et al. (2021) [33]	Post elimination	2014–2020	Spain	14
Richard et al. (2009) [39]	Endemic	2006–2009	Switzerland	7
Risco–Risco et al. (2017) [34]	Endemic	2003–2014	Spain	3
Pacenti et al. (2019) [18]	Endemic	2017–2018	Veneto, Italy	3
Bianchi et al. (2022) [17]	Endemic	2017–2021	Lombardy, Italy	8

Nonetheless, measles infection in vaccinated individuals is usually associated with an intense and/or prolonged exposure to an infected individual. For example, prolonged exposure to an acutely ill patient in a medical setting has resulted in several reports of measles reinfections among health care workers (HCW) [40]. A study conducted in Spain in the post-elimination era reported that around 27% of the measles cases occurred in fully vaccinated subjects acquired the infection while working in a healthcare setting [33]. Rota et al., described measles in two fully vaccinated physicians after treating MV-positive patients during the 2009 measles outbreaks in Pennsylvania and Virginia [41]. In the western Netherlands, in 2014, two fully vaccinated and one HCW vaccinated with a single dose exposed to two hospitalized MV-positive cases developed measles [42]. Transmission in health care setting has been observed also between patients. In 2019, Hubiche et al., described a measles outbreak in a teenage psychiatric unit with high vaccination coverage in France. The index case was a 16-year-old unvaccinated female who transmitted MV to two fully vaccinated subjects. None of the patients’ contacts in the unit (at least 19) developed measles. The authors suggest that measles transmission within patients was facilitated by the close contacts between them in the therapeutic group [43]. Furthermore, measles transmission to vaccinated individuals has been reported in familiar setting. For example, de Oliveira et al., described MV transmission to a 19-year-old fully vaccinated female living with her unvaccinated uncle, who tested positive to measles [44].

## 4. Vaccine Failure Classification

There are two major factor categories associated with vaccine failures: vaccine-related and host-related factors. Vaccine related factors include incomplete attenuation, incorrect immunization route or schedule, and interruption of the cold chain (attenuated measles vaccines lose potency if not properly stored at 2–8 °C after reconstitution). Host-related factors include host genetics, immune status, age, and health or nutritional status [45]. Single-nucleotide polymorphisms of cytokine and cytokine receptor genes and genetic variants of genes involved in MV infection, inactivating mutations in the type I interferon receptor IFNAR1, the high-affinity interferon α/β receptor IFNAR2, and the transcription factors signal transducer and activators of transcription (STAT) 1 and STAT2 could influence the effectiveness of the response to vaccination [18].

**Figure 2:**
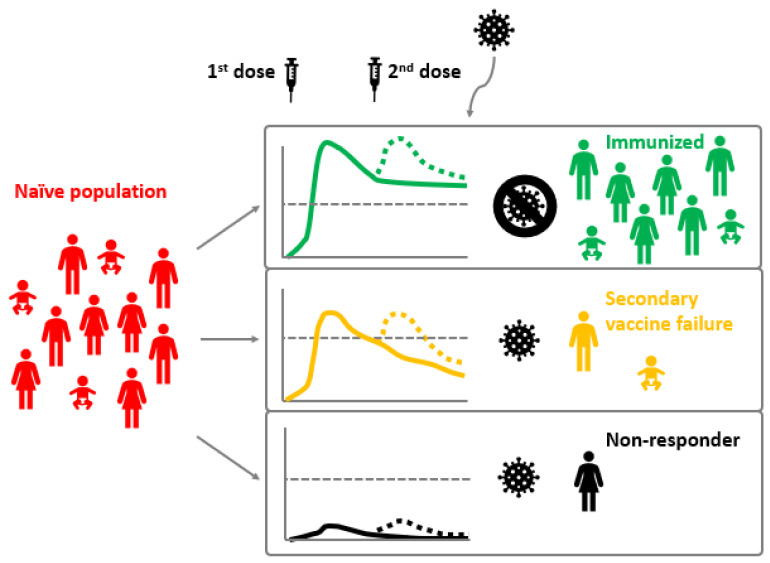
Possible vaccine outcomes in relation to type G immunoglobulins (IgG) levels. Immunization with a first vaccine dose causes the development of an immune response and the production of long-lasting levels of IgG (continuous lines) while a second vaccine dose causes a boost of IgG levels (dotted lines) that increases protection duration. Successfully immunized individuals maintain, already after the first dose, or develop after the second dose, an immunity that protects them from future infections and IgG levels are above the protective level (grey lines). Secondary vaccine failure occurs when the level of IgG drops below the protective level (although IgG can still be detected in these individuals), while non-responders are those subjects that never develop protective immunity (unmeasurable IgG levels).

These factors can lead to primary or secondary vaccine failures. Primary vaccine failure is the complete absence of a measurable humoral immune response [45,46]. Subjects that fail to seroconvert after the vaccination are also referred to as non-responders. Secondary vaccination failure consists of a sub-optimal or non-protective response to immunization by the vaccination or in the loss of vaccine-induced immunity over time [46,47,48] (Figure 2).

**Figure 3:**
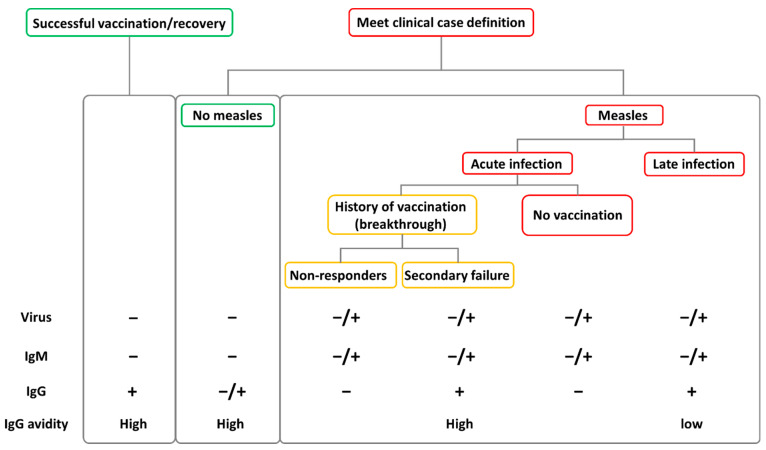
Graphical representation of molecular and serological profiles that can be detected during diagnostic tests in different cases after MV infection compared to non-infected subjects. A primary infection usually causes the development of both IgM, which disappear in later stages of the infection, and long-lasting IgG. When a case meets the clinical definition of measles the presence of IgM and/or the detection of the virus indicates the occurrence of an active acute infection (right panel), while their absence implies a different infection causing the symptoms (middle panel). During an acute infection, a non-immunized subject will not yet present measurable IgG while, in a post-acute infection (beyond 10 days), low-avidity IgG (indicating a recent infection) will be measurable in these subjects (while IgM and virus levels drop) (red boxes in the right panel). During reinfection, IgG would not be measurable in non-responders while high-avidity IgG (indicating a past infection) can be detected in secondary vaccination failure (yellow boxes in the right panel). During a post-acute reinfection non-responders can present low-avidity IgG. A recovered or successfully vaccinated individual will present no acute infection markers (IgM and virus) while possessing high-avidity IgG (left panel).

The best method to differentiate breakthrough infections is the presence or absence of type G immunoglobulins (IgG), which develop later during the infection and persist for long periods of time, and the avidity enzyme immunoassay, which allows to differentiate a recent (low avidity IgG) from a past (high avidity IgG) infections [49] (Figure 3). During the acute phase of a breakthrough infection, non-responders are recognized by the absence of IgG or, during a post-acute stage of the infection (beyond 10 days), by the presence of IgG of low avidity as the only antibodies detectable in these individuals are those produced during the reinfection. Individuals with a secondary vaccination failure are characterized by the presence of high-avidity IgG during the acute infection phase as these patients possess IgG from past infections that, however, did not protect them from a reinfection. Both non-responders and secondary vaccine failure cases can develop IgM during the reinfection. Notably, subjects with low post-vaccination antibody titers could still maintain protective antibody levels and have an adequate response against MV infection [46].

The distinction between the two types of vaccine failure is critical as it helps understanding the causes behind outbreaks in highly vaccinated populations and the role of vaccine failures in MV spread, especially in a measles elimination goal setting [50].

Primary vaccination failure has been observed in around 5% (range 2–7%) [51] of measles vaccinees and for this reason two doses of measles vaccine are recommended [52]. Indeed, approximately 95% of the subjects that does not respond to the first vaccine dose responds to a second dose [53,54]. Maternal antibodies inhibitory effect and immunological maturity of the vaccine recipient are the most common causes of primary vaccine failure [54]. Analysis of the number of vaccine doses and the type of vaccine failure showed a higher primary vaccine failure rate in individuals who received two doses before one year of age compared with those who received the two doses later [48]. During the 1997 São Paulo Epidemic, Pannuti et al., reported a significantly higher percentage of primary vaccination failure cases in individuals who had received a single dose of measles vaccine (69.7%) than in fully vaccinated subjects (12.5%) [48]. The same was observed by Atrasheuskaya et al., in a study conducted in Novosibirsk between 2000 and 2005 (71% of non-responders after a single dose vaccinees vs. 38% among fully vaccinated) [55].

It is estimated that secondary vaccination failure occurs in 2–10% fully vaccinated individuals [17,42,48,55,56,57,58,59]. Measles surveillance activities conducted in Milan between 2017 and 2021 highlighted that, of the 653 measles confirmed cases, 25 were fully vaccinated (3.8%). Secondary vaccination failures encompassed 48% of fully vaccinated subjects (12/25) [17]. On the other hand, during an outbreak in a rural community close to Vancouver, Mathias and colleagues reported a secondary vaccination failure rate of 5% in a cohort of 175 children mostly immunized with a single vaccine dose [57].

## 5. Clinical Manifestations of Breakthrough Cases and Diagnostic Challenges

Different studies recognized a milder disease in breakthrough cases (modified measles), especially in secondary vaccination failure cases and in fully vaccinated individuals. Indeed, despite sometimes insufficient immunization to adequately protect against the infection develops, measles vaccination still limits the risk of complicated measles [43]. However, these subjects do not always present with signs and symptoms typical of measles, making the clinical diagnosis more challenging [32,49]. For instance, rash may not follow the usual progression, the initial sites may be the trunk and arms rather than the face, and the infection may result in a full body rash [40]. Fully vaccinated secondary vaccine failure cases are less likely than unvaccinated patients, those vaccinated with only one dose, or non-responders to have cough, coryza, conjunctivitis and fever [32,35,60] and/or to be hospitalized [17,18,32,61], demonstrating a protection against the severe forms of the disease. For example, Helfand et al. described an outbreak occurred in an immunized population exposed to MV during a bus trip and observed how none of the 44 individuals exposed to the index cases and participating in the study showed an illness that met the CDC clinical definition. In this outbreak, only 22 passengers experienced at least one of the following symptoms: conjunctivitis (5 subjects), coryza (6), cough (15), diarrhoea (5), fever (10), headaches (15), joint aches (3), swollen lymph nodes (6), photophobia (5), rash (1), sore throat (13), or vomiting (1) [62]. Coleman et al., observed a milder disease (fever, mild cough, conjunctivitis, and mild rash) in two fully vaccinated siblings exposed to a MV-positive passenger during a south-west China to Melbourne flight compared to an unvaccinated passenger of the same flight [63]. Sheppeard et al., during an outbreak occurred in New South Wales in 2016, observed that the six MV-positive children, who had a confirmed history of vaccination with at least one dose of MMR, showed a lower number of symptoms compared to the other 27 unvaccinated cases and presented with an atypical or no rash and a shorter prodrome. Furthermore, no vaccinated children were hospitalized [64]. Rota et al. described the atypical manifestation of MV infection in two fully vaccinated physicians. One of the physicians reported flu-like symptoms, including myalgia, cough, and fever that lasted four-five days prior to the appearance of a rash that spread from the abdomen to the neck and resolved within 24 h. The other physician reported fever, headache, and rash, but no coryza, conjunctivitis, or cough [41]. During a measles epidemic in 1997 in Niterói, Brazil, de Oliveira et al. described measles transmission from an unvaccinated male to his vaccinated niece. While the male developed an illness that could be clinically and serologically diagnosed as measles, the female did not develop IgM and showed a modified measles disease course and an increase in MV-specific IgG in serum. In both cases, measles infection was confirmed through RNA detection by reverse transcriptase polymerase chain reaction (RT-PCR). The index case presented a 5-day history of high fever (39.5 °C), cough, coryza, conjunctivitis, diarrhoea, Koplic spots and a maculopapular rash lasting for two days. He recovered completely in 10 days. The vaccinated cases showed high fever for two days, myalgia and a maculopapular rash only on face and neck that lasted for 1 day. She recovered completely within 4 days [44]. If typical signs and symptoms of measles are present, they often resolve quickly [40]. Primary vaccination failure cases, despite being less likely to be hospitalized compared to unvaccinated cases [17,33], are more likely to display typical measles symptoms [33,40].

Because of the milder disease, the identification of secondary vaccination failure cases based only on clinical features is unreliable [40]. Patients with mild measles are less likely to seek medical attention and providers may be less likely to perform tests for measles in vaccinated subjects. Therefore, it is likely that secondary infections occur more frequently than reported and this could be critical in light of the global measles control strategies. To overcome this issue, in a measles elimination setting, a highly sensitive case-based surveillance is essential for the timely detection of cases/outbreaks and to accurately define the extent of susceptible people and populations [10]. In a study conducted in Spain in the post elimination era, half of the measles cases that occurred in fully vaccinated subjects could have gone undetected because of the lack of the classical set of symptoms that usually trigger surveillance activities [33]. Therefore, the best methods to confirm MV infection in these cases are the detection of MV RNA in oropharyngeal swabs or urine samples by real time RT-PCR, even if the window for RNA detection may be shorter than that of a primary measles case [40], combined with a full serological profiling. Indeed, since IgM antibodies may not be produced, serology could be useful to confirm secondary vaccine failure cases, which usually show in their sera collected during the acute phase highly reactive IgG antibodies of high avidity, consistently with a prior immunological response to MV [40] (Figure 3).

## 6. Onward Transmission from Breakthrough Cases

Although rare, transmission from vaccine failure cases is possible. Several studies reported no evidence of transmission from secondary vaccine failure cases [38,41,42,65,66], while others reported onward transmission from both primary and secondary vaccination failure cases [17,32,35,58,60,67], with vaccinated people acting both as index cases as well as secondary transmitters [17,58,60,61] (Table 2). These conflicting data suggest that onward transmission from vaccinated cases to susceptible individuals is limited to specific settings where close contacts are more common, such as familiar or nosocomial environments. The low rate of transmission from breakthrough cases may be associated to the elevated and rapid production of neutralizing antibodies that quickly reduce the viral load, but also to the manifestation with milder symptoms (i.e., mild, or unproductive cough) that reduces the likelihood of an effective transmission of the virus [40]. Nevertheless, the fact that a MV infection and onward transmission can both occur for subjects with vaccine failures, underscores the need to maintain a high index of suspicion for measles during an outbreak and to monitor all subjects despite (presumed) prior vaccination or disease [61]. Specifically, the same effort in tracing contacts should be dedicated to identifying possible infections amongst vaccinated and unvaccinated subjects and vaccinated cases should also carefully follow virus containment procedures. This is of particular importance in health care settings.

Nonetheless, since secondary infections seem to be overall less transmissible than primary infections, it is believed that they do not significantly affect measles eradication plans [58]. This is consistent with the observation of sustained measles elimination in populations with high vaccination coverage, including those where many individuals were vaccinated more than 40 years earlier [52].

Measles cases in vaccinated individuals are generally characterized by low viral RNA loads in bodily fluids compared with primary infections. The lower levels of viral genome copies may explain the limited infectiousness of breakthrough infections, and this is in line with numerous studies that indicate that breakthrough infections rarely cause onward transmission [41,42,66,68]. Various studies reporting outbreaks in vaccinated populations have used cycle threshold (Ct) value as a proxy to semiquantitatively measure the viral load in biological samples. Fewer Ct values indicate higher numbers of viral RNA copies in a sample. Pacenti and colleagues described the characteristics of 11 secondary vaccine failure cases occurred in Veneto Region, Northern Italy [18]. In these patients, measured MV RNA Ct values in nasopharyngeal swabs and in urine were significantly higher compared with a control group of forty unvaccinated subjects, indicating lower viral loads. Data obtained by Sundell et al. [35] indicate that individuals with breakthrough infections involved in an outbreak in Gothenburg, Sweden, presented lower levels of measles RNA in nasopharyngeal samples compared with primary infections. In this study, the authors hypothesized that the large difference in viral load could be explained by the fact that a partial immunity could reduce viral replication. Bianchi and colleagues, in a detailed analysis of breakthrough measles cases in Milan, Northern Italy [17], showed significantly higher Ct values in oropharyngeal swabs and urine in breakthrough cases compared to those with no vaccination history. In addition, the authors described lower Ct values for patients with primary vaccination failure compared with patients with secondary vaccination failure. Seto and colleagues recently described a large measles outbreak in Yamagata Prefecture, Japan, and analyzed Ct values for throat swab specimens to estimate the infectiousness of cases. The authors performed three serological assays (plaque reduction neutralization, IgG avidity, and gelatin particle agglutination) on samples collected from 31 patients with measles and coupled the results with the Ct values obtained from the analysis of respiratory specimens. This revealed that respiratory samples of patients with low antibody titers had low Ct (i.e., high viral load), while non-spreaders tended to show low viral loads [68].

## 7. Booster Doses and Catch-Up Vaccination

Several studies estimated that breakthrough infections occur between six and twenty-six years after the last measles vaccination [17,18,32,33,38,55]. A progressive decrease in levels of anti-MV antibodies as time since vaccination increases has been observed, as also shown by a prospective cohort study performed in the United States [30]. LeBaron and colleagues, in a study conducted in schoolchildren in a post elimination environment, observed a decrease in neutralizing antibodies ten years after vaccination, with 4.7% of fully vaccinated children considered potentially susceptible to reinfection (neutralizing titres lower than 120 mIU/mL) [30]. The analysis conducted by Pacenti et al., showed that in over 90% of subjects, antibody titres remained above the level of protection up to 30 years after vaccination [18]. These results were consistent with previous studies [69]. Therefore, even if the percentage of susceptible individuals several years after vaccination seems to be low, population immunity to MV should be monitored, especially in adult age groups, to assess potential declines of protection.

However, waning of measles antibody titres does not necessarily correspond to a waning immunity, since cell-mediated responses are also known to play an important role in protection [65]. Ovsyannikova et al., showed that measles immunization promotes the development of MV-specific memory T-cells that persist for decades after the immunization [70]. On the other hand, Lin and colleagues demonstrated that the vaccine-induced T-cell response contributes to a faster clearance of viral RNA after infection but did not prevent the occurrence of the infection [71].

Furthermore, it has been observed that, although secondary vaccine failure cases lacked sufficient protective neutralizing antibody levels to completely inhibit MV infection at the time of exposure, they still rapidly mounted a remarkable neutralizing response that likely mitigated extensive viral replication and resulted in a mild disease with minimal symptoms and fewer complications [65].

The duration of MMR vaccine-induced immunity in the absence of circulating virus is still to be completely elucidated and it may be significantly impacted by the age at first vaccination, as well as by the timing of the second dose. Consistent implementation of a two-dose schedule is also needed to maintain high population immunity against MV, to minimize disease, and to prevent subsequent outbreaks [32].

Catch-up vaccination is recommended for all individuals (children, adolescents, and adults) who have not received the first or second dose of MV vaccine. However, in many European countries catch-up vaccination programs are not efficiently conducted or well accepted. Consequently, measles outbreaks are still occurring despite significantly increasing vaccination rates, with many adolescents and young adults (up to 40 years of age) being affected [72]. Furthermore, during 2020, childhood immunization services have been disrupted by the COVID-19 pandemic in about 70 countries, with around 80 million children being affected [73]. Reduced routine vaccination coverage without catch-up vaccination may lead to an increase in measles burden worldwide. The effect of lower measles vaccine coverage has not yet resulted in an increase in the number of cases and deaths, probably because prevention and control measures introduced to reduce the spread of SARS-CoV-2 have also reduced the spread of MV [74]. Nonetheless, it is crucial to implement catch-up vaccination campaigns and close this immunization gap before the consequences of the reduced coverage will start manifesting.

## 8. Conclusions

All six WHO regions have established or expressed a commitment to achieving regional control or elimination of measles, although the targets and milestones on the path to elimination vary between regions [10]. To achieve the elimination goal, high levels of immunity and a low proportion of breakthrough infections are needed, while the immune status of the population and the circulation of the virus (including molecular typing) needs to be constantly monitored. Still, the highest number of measles cases occur in non-vaccinated subjects, and increasing vaccination coverage is pivotal to achieve elimination. However, as vaccination coverages increase, the number of breakthrough infections is expected to grow, and it is essential that we acquire a deeper understanding of the atypical clinical manifestations of these cases and learn to detect and quantify them if we want to be able to properly monitor MV epidemiological trends and be prepared to handle potential future outbreaks. Nonetheless, despite the occasional occurrence of measles in vaccinated individuals, breakthrough infections do not seem to be a direct threat for measles elimination, and it is unlikely that the level of population immunity will drop below herd immunity thresholds in a post elimination setting. However, although rarely, spreading from breakthrough cases does occur and vaccine failure cases do play an important role in disease spread and outbreak management [50] and need to be properly identified so that appropriate control measures to reduce onward transmission can be implemented [50]. The implementation of sensitive surveillance systems coupled with full case-based laboratory examinations are, therefore, the two crucial aspects that will allow for prompt identification and accurate monitoring of breakthrough measles cases as vaccination coverage increases, and measles elimination programs move forwards.

## Figures and Tables

**Table 2:** Measles onward transmission after secondary infections.

Study	Evidence/No Evidence of Transmission
**No evidence of transmission from vaccine failure cases**	
Rota et al. (2011) [58]	No evidence of transmission from two MV positive fully vaccinated physicians. More than 100 patients and close familiar contacts were exposed, but no additional cases were identified.
Hickman et al. (2011) [65]	No evidence of transmission from eight individuals with primary or secondary vaccine failure.
Jones et al. (2015) [66]	No evidence of measles transmission from a vaccinated nurse that tested positive in both molecular and serological tests. A total of 71 vaccinated HCW were exposed to the nurse and 478 patients and family members were potentially exposed.
Sundall et al. (2015) [35]	No identification of onward transmission from 16 measles breakthrough cases.
Hahné et al. (2016) [42]	No evidence of measles transmission from seven immunized HCW despite they travelled abroad, used public transportation, and worked at the hospital.
Augusto et al. (2019) [38]	No secondary cases generated by ten vaccinated individuals infected during two different outbreaks in Portugal.
**Evidence of transmission from vaccine failure cases**	
Edmonson et al. (1990) [60]	A fully vaccinated high school student with serologically confirmed measles transmitted the virus to 13 previously vaccinated classmates and gave rise to a measles outbreak involving 218 cases.
Rosen et al. (2014) [58]	Report of an outbreak of five measles cases in New York City during which a fully vaccinated index patient with documented secondary vaccine failure transmitted MV infection to four contacts with documented vaccination or prior positive anti-MV IgG antibody tests. A total of 88 individuals were exposed to the index cases and an additional 231 contacts were identified as exposed to the secondary patients. No tertiary cases were identified among these contacts.
Santibanez et al. (2014) [67]	Evidence of MV transmission from a secondary vaccine failure case within a family after MV spread initiated at an international mass gathering.
Cherry et al. (2018) [32]	Evidence of transmission from three vaccine failure cases with ≥2 doses to household contacts or close friends.
Bianchi et al. (2022) [17]	Evidence of onward transmission for ten vaccinated subjects. In eight outbreaks vaccinees were the index case and transmitted MV to two to four people.
